# Activity Begins in Childhood (ABC) – inspiring healthy active behaviour in preschoolers: study protocol for a cluster randomized controlled trial

**DOI:** 10.1186/1745-6215-15-305

**Published:** 2014-07-29

**Authors:** Kristi B Adamo, Nick Barrowman, Patti Jean Naylor, Sanni Yaya, Alysha Harvey, Kimberly P Grattan, Gary S Goldfield

**Affiliations:** Children’s Hospital of Eastern Ontario Research Institute (CHEO-RI), 401 Smyth Road, Ottawa, ON K1H 8 L1 Canada; Healthy Active Living and Obesity Research Group (HALO), 401 Smyth Road, Ottawa, ON K1H 8 L1 Canada; School of Exercise Science, Physical and Health Education, University of Victoria, PO Box 3015, STN CSC, Victoria, BC V8W 3P1 Canada; Faculty of Medicine, Pediatrics, University of Ottawa, 451 Smyth Road, Ottawa, ON K1H 8M5 Canada; Faculty of Health Sciences, University of Ottawa, Ottawa, ON K1N 7 K4 Canada

**Keywords:** Childcare, Daycare environment, Intervention, Motor skills, Paediatric health, Physical activity, Preschool, Sedentary behaviour

## Abstract

**Background:**

Today’s children are more overweight than previous generations and physical inactivity is a contributing factor. Modelling and promoting positive behaviour in the early years is imperative for the development of lifelong health habits. The social and physical environments where children spend their time have a powerful influence on behaviour. Since the majority of preschool children spend time in care outside of the home, this provides an ideal setting to examine the ability of an intervention to enhance movement skills and modify physical activity behaviour. This study aims to evaluate the efficacy of the Activity Begins in Childhood (ABC) intervention delivered in licensed daycare settings alone or in combination with a parent-driven home physical activity-promotion component to increase preschoolers’ overall physical activity levels and, specifically, the time spent in moderate to vigorous physical activity.

**Methods/design:**

This study is a single site, three-arm, cluster-randomized controlled trial design with a daycare centre as the unit of measurement (clusters). All daycare centres in the National Capital region that serve children between the ages of 3 and 5, expressing an interest in receiving the ABC intervention will be invited to participate. Those who agree will be randomly assigned to one of three groups: i) ABC program delivered at a daycare centre only, ii) ABC program delivered at daycare with a home/parental education component, or iii) regular daycare curriculum. This study will recruit 18 daycare centres, 6 in each of the three groups. The intervention will last approximately 6 months, with baseline assessment prior to ABC implementation and follow-up assessments at 3 and 6 months.

**Discussion:**

Physical activity is an acknowledged component of a healthy lifestyle and childhood experiences as it has an important impact on lifelong behaviour and health. Opportunities for physical activity and motor development in early childhood may, over the lifespan, influence the maintenance of a healthy body weight and reduce cardiovascular disease risk. If successful, the ABC program may be implemented in daycare centres as an effective way of increasing healthy activity behaviours of preschoolers.

**Trial registration:**

Current Controlled Trials: ISRCTN94022291. Registered in December 2012, first cluster randomized in April 2013.

## Background

The prevalence of child obesity has increased dramatically over the past three decades [1–3] and attenuating these rates is a high priority in Canada, not only from a population health perspective, but from the health care system’s economic perspective. Moreover, obesity tracks very closely from childhood to adolescence to adulthood [2, 4]. Six in ten obese children have at least one risk factor for cardiovascular disease, and an additional 25% have two or more risk factors [4]. Co-morbidities, such as Type 2 diabetes and non-alcoholic fatty liver disease, once considered problems among adults, are now being reported at a greater frequency among youths [5–8]. The greater risk of health complications associated with early morbidity affects normal childhood development and quality of life. In addition, the long-term health care burden increases exponentially if we include the obesity-associated chronic co-morbid conditions. It has been projected that the current generation of children will be the first in modern history to see a shorter life-expectancy than their parents [1] and we know that once it has developed, obesity is very difficult to treat. Indeed, there are many critical periods for intervention over one’s lifespan; however, these findings underscore the importance of prevention early in life and recent mathematical modelling suggests that targeted interventions for young children (0 to 6 years) could yield considerable cost savings to the health care system [9].

Canadian surveillance data, using directly measured heights and weights gathered as part of the Canadian Community Health Survey, indicate that overweight and obesity exist in the preschool age group with 15.2% of children aged 2 to 5 years categorized as overweight and 6.3% as obese [3]. Overweight children have higher risks for numerous health conditions and children who become obese before the age of 6 years are likely to be obese later in childhood [2]. The negative trajectory continues as these children often remain overweight as adults [4]. Physical inactivity is associated with increased risk of several chronic diseases including obesity and heart disease [5, 7] and we know that only 7% of Canadian children between the ages of 6 and 19 years are meeting current physical activity (PA) guidelines [8]. There is considerable evidence to indicate that reduced PA or increased sedentary behaviour are implicated in the etiology of childhood obesity and its associated conditions [6, 9, 10]. From the cardio-metabolic standpoint, the currently available evidence, albeit sparse, indicates that PA during the preschool years is associated with i) more desirable body composition variables [11–16] and ii) decreased cardiovascular risk factor status (i.e., lower total cholesterol [17], higher HDL cholesterol [17, 18], and lower sub-maximal heart rate during exercise [19]). Furthermore, motor development, or the process by which a child acquires movement patterns and skills, has also been shown to be positively associated with PA [20–22]. Early motor development is important as motor skills are a key factor in the likelihood of participation in various forms of PA during later childhood and adolescence [21, 23, 24].

PA is an acknowledged critical component of a healthy lifestyle and childhood experience as it has a meaningful impact on lifelong behaviour and health. Opportunities for PA and motor development in early childhood may, over the lifespan, influence the maintenance of a healthy body weight and reduce the risk of cardiovascular disease. It has been noted that children with low movement competence usually exhibit low PA levels [25, 26] and tend to be vigorously active less often, play less on large playground equipment, and spend less time interacting socially with their peers [25]. Fundamental movement skills (e.g., catching, throwing, jumping, and running) are the essential building blocks for the acquisition of more refined and complicated skills that can be applied later in life, such as sporting, recreational, and physical activities [27–29]. However, movement skills will not develop to their full potential without opportunities to practice in environments that are stimulating and supportive [30, 31]. Butcher and Eaton [26] found that preschoolers’ movement competence was already influencing their PA levels and their PA choices.

### Rationale

There is a paucity of information on current trends in PA of preschool aged children in Canada, [32–34] and on the relationship between the ability of children to perform fundamental movement skills and prediction of PA [35]. A 2012 systematic review was recently performed by Timmons et al. [36] to assemble and interpret the best available evidence for minimal and optimal amounts of PA needed to promote healthy growth and development in young children, including preschoolers. The underlying objective of this review was to help inform the development of evidence-based PA guidelines for this age group. The subsequently published guidelines recommend that “*preschoolers (aged 3–4 years) should accumulate at least 180 min of physical activity at any intensity spread throughout the day, including a variety of activities in different environments, activities that develop movement skills, and progression toward at least 60 min of energetic play by 5 years of age*” [37]. A similar review [38] has also been published advocating that preschool-aged children should not be sedentary for more than 60 minutes at a time (less is better), except when sleeping, and guidelines have since been published [39] to this effect.

More than half of Canadian children between the ages of 6 months and 5 years are enrolled in some form of non-parental care, with a mean of 29 hours per week in this arrangement [40, 41]. Canada fails miserably on an international scale, in comparison to all other Organisation for Economic Co-operation and Development countries, with regard to treatment of our youngest and arguably most vulnerable citizens. According to the UNICEF Report Card, entitled *The Child Care Transition*, which focuses on the shift from parents raising children to out-of-home daycare, Canada was tied for last place in ensuring that this age group is getting high quality out-of-home care [42]; the key criticism of Canada was “*lack of substantial public investment in education until children reach the age of 5*”. This is a critical issue given that Canadian children are now spending more time in care outside of the home than ever before. Recognizing that the landscape of childcare in the developed world has changed dramatically over the last two decades, with the vast majority of children now attending some form of daycare during their early years, the preschool environment represents a focal point with great promise for health interventions. Several groups throughout the world have similarly identified the need to encourage and support PA within the preschool curriculum [43–45] and various teams are working towards evaluating potential solutions [46–51]. Uniquely, this Canadian three-arm RCT study is poised to explore not only changes in PA and anthropometrics, but motor skill development and quality of life, as well as changes to the daycare environment and to identify if the addition of a home component results in greater benefits.

We know that i) physical inactivity and poor fitness are independent risk factors for obesity, metabolic disorders, and cardiovascular disease in youth, ii) successful development of motor skills provides stimulus for ongoing PA engagement contributing to long-term health [52], and iii) PA levels track from early childhood to adulthood. Consequently, increasing children’s PA levels in the preschool years may alter their activity trajectory and increase the likelihood they will be physically active throughout development stages and into adulthood. The proposed intervention, Activity Begins in Childhood (ABC), is clinically relevant as it includes training workshops for daycare-providers focusing on the importance of PA and reducing sedentary behaviour, as well as strategies for implementing a variety of structured and unstructured physical activities to meet these objectives. This study will be able to address how viable the daycare setting is to promote PA in preschool-aged children and the possible incremental value of the home setting.

## Methods/design

### Study objectives

The primary objective of this study is to evaluate the efficacy of the ABC intervention program delivered in licensed daycare settings alone (intervention, daycare (DC)) vs. standard daycare curriculum (comparison (COM)) to increase preschoolers overall PA levels, specifically time spent in moderate to vigorous physical activity (MVPA).

The secondary objectives aim:

-  To evaluate the potential additive contribution of a parent-driven home PA-promotion in addition to the daycare provider-facilitated intervention on its own (i.e., intervention DC vs. DC + HOME)
-  To evaluate the efficacy of the ABC intervention arms to decrease the amount of time spent in sedentary behaviour
-  To evaluate the effects of the ABC intervention arms on fundamental and gross motor skills in preschoolers attending daycare centres
-  To evaluate the effects of the ABC intervention arms on preschool children’s anthropometrics, such as height, weight, body mass index, lean body mass, fat mass, and percent body fat
-  To assess the effects of the ABC intervention on daycare providers’ attitudes, control beliefs, perceived competency, and intentions toward incorporating PA into the daycare curriculum, and examine whether these social-cognitive variables impact PA in children
-  To evaluate the effects of the ABC intervention on child’s quality of life and on the quality of life of the parents as well
-  To evaluate the costs of delivering the ABC intervention with the outcomes achieved to provide a valuable analytical framework to guide decision making by those who are responsible for allocating resources

### Hypotheses

We hypothesize that MVPA (minutes/day) will be greater in the combined DC + HOME arm when compared with the DC-only arm, but that MVPA will be greater in both intervention groups vs. the COM group. We believe both intervention arms will show larger reductions in sedentary behaviour and greater improvements in fundamental and gross motor skills, body composition, and quality of life at the 6-month follow-up compared to the COM arm, with the combined intervention (DC + HOME) being superior. We predict that the ABC intervention will enhance providers’ and parents’ attitudes, control beliefs, perceived competency, and intentions to increase children’s PA in the daycare setting and home environment immediately following the training workshop, and consistent with the theory of planned behaviour, these process measures will predict children’s PA at follow-up.

### Trial design

**Figure 1:**
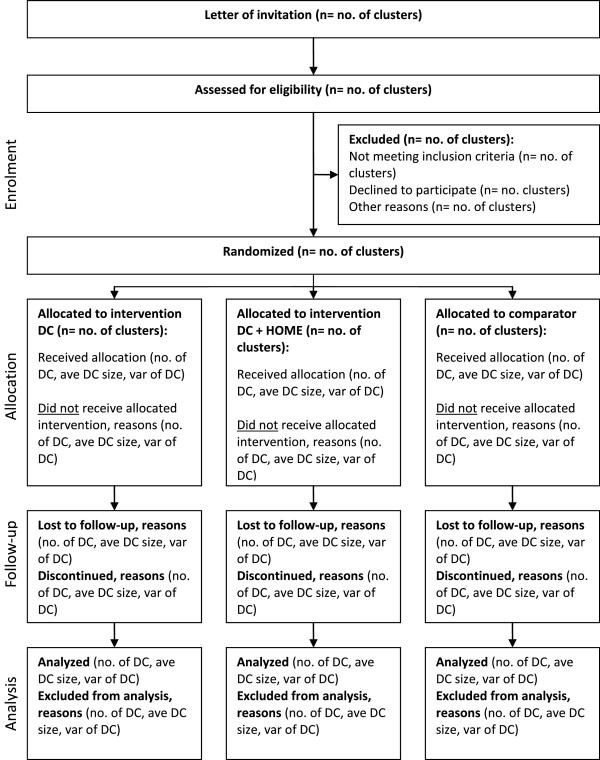
**CONSORT flow diagram.**

We are carrying out a single site, three-arm, cluster-randomized controlled trial design in Canada’s National Capital Region, with daycare centres as the unit of measurement (clusters) to evaluate the efficacy of the ABC intervention and training manual to increase PA (Figure 1).

### Participants, inclusion/exclusion, and recruitment

#### Daycare centres

All licensed daycare centres in the National Capital Region (NCR) that serve children between the ages of 3 and 5, with an enrolment of >20 children in this age range, and provide service in either English or French, are eligible to participate. Daycare recruitment will commence with an initial letter mailed out to the directors of all daycare centres within the NCR, requesting that the directors contact the research coordinator for further information related to the study if they are interested in taking part. Daycare directors who agree for their site to participate in the trial are committing to modify their curriculum accordingly and support their staff in facilitating the required changes if they are randomized to the intervention arm and control groups must agree to allow study personnel to visit their facilities for planned assessments.

#### Preschoolers

All preschool children between the ages of 3 and 5 years attending the involved daycare centres, who plan to remain within the respective daycare setting for at least 6 months, and whose parents speak either English or French, will be eligible to participate regardless of mental or physical capabilities. While the daycare environment will be subjected to the intervention, only children whose parents sign informed consent will be assessed.

### Ethics and consent

The study has been approved by the Research Ethics Board at the Children’s Hospital of Eastern Ontario Research Institute (REB protocol number: #12/158X). The daycare centres which meet the study inclusion criteria will be sent an information sheet and asked to indicate their desire to participate by providing written consent to the research coordinator. Following daycare director consent, parents of the potential participants will be approached via letter, poster, and word of mouth. Interested participants will provide a letter of informed consent to the research coordinator. Participants involved with the home intervention and daycare providers involved with the intervention will be reimbursed for their time with a $50 (Canadian dollars) gift certificate to a local grocery store chain.

### Randomization

Eighteen daycare centres (clusters) that agree to participate will be randomly assigned to one of three groups: i) ABC program delivered at daycare centre only (n = 6; DC), ii) ABC program delivered at daycare plus a home component (n = 6; DC + HOME) or, iii) regular daycare curriculum (n = 6; COM).

Because daycare centre size, language of delivery, and season may influence the outcomes of interest, randomization will be stratified based on these variables. Small-medium daycare centres will be defined as those serving 10 to 20 children in our 3- to 5-year-old targeted age range, those enrolling 21 or more preschoolers will be considered large. To take into account possible seasonal effects on PA, the trial will be stratified by season: 8 sites will be randomized in the Spring of 2013 (3 in DC, 3 in DC + HOME, and 2 in COM)), 7 in the Fall of 2013 (2 in DC, 2 in DC + HOME, and 3 in COM), and 3 will be randomized in the Fall of 2014 (1 in DC, 1 in DC + HOME, and 1 in COM). The randomization sequence will be computer generated by a data manager at the Clinical Research Unit at the Children’s Hospital of Eastern Ontario, who is not affiliated with this trial. Before running the randomization program, the data manager will document the study ID and daycare centre size, and season. After running the program, the data manager will document the group assignment and then inform the study coordinator of the assignment. To ensure adequate allocation concealment, the randomization sequence list will be kept centrally with the data manager at the Clinical Research Unit. It will not be accessible to members of the study team and subjects and only be activated when a centre is eligible to be randomized.

#### ABC intervention (common to DC and DC + HOME)

The ABC intervention, designed to increase PA and reduce sedentary behaviour, will consist of two 3-hour workshop training sessions conducted by a master trainer with experience in promoting PA in preschoolers. The training workshops will target the daycare providers of 3- to 5-year-old children assigned to both the DC and DC + HOME arms of the intervention trial. The first workshop will focus on the importance of PA and movement skills for preschool-aged children, understanding structured and unstructured play, familiarization with the intervention tools and broad practicalities of implementing ABC in daycare centres, and include demonstrations of activities related to movement skills. The second workshop will focus on the goals of i) providing opportunities for light, moderate, and vigorous PA for at least 15 minutes per hour while the children are in care, ii) providing daily outdoor time for PA when possible, iii) providing a combination of developmentally appropriate structured and unstructured PA experiences, iv) becoming an active participant and joining children in PA, as well as providing verbal guidance and encouragement, v) integrating PA into activities designed to promote cognitive and social development, vi) providing both indoor and outdoor environments with a variety of portable play equipment and adequate space per child, vii) avoiding the punishment of children for being active, and viii) avoiding disciplining/punishing children by withholding PA. The training will work on overcoming barriers to facilitating PA, understanding the range of movement skills, and using everyday materials to facilitate PA and active play.

In addition, each daycare centre in the intervention groups will be given the ABC resource training manual, recommended activity program outline with log sheets, music developed for PA with an outlined guidebook, and a starter kit of equipment that will form the basis of training for the daycare providers and the intervention itself. The manual is compiled with various ways providers can get children active in structured and unstructured physical activities, some of which target motor skill development. Our team will provide the educators with weekly schedules that suggest a set of activities, drawn from the ABC manual, that can be incorporated into the daily curriculum with the aim of meeting the Canadian Society for Exercise Physiology guidelines of accumulating at least 180 minutes of PA at any intensity spread throughout the day for preschool-aged children [37] and at least 60 minutes of MVPA for the children aged 5 years [53]. Additionally, these guidelines also suggest limiting the children’s sedentary time and minimizing screen time to less than 60 minutes a day for children aged 3 to 4 years, and limit recreational screen time to less than 2 hours for children aged 5 years [54]. These suggested activities have been broken down as locomotor skills, manipulative motor skills, and moving to music, shapes, and creative play. The program includes 60 minutes of structured activities, with the largest percentage (50%) of time spent on locomotor skills, as those are the higher intensity activities (such as running to play tag) and will more likely increase MVPA. Manipulative motor skill activities aid in developing gross motor skills which are important for major body movement such as walking, maintaining balance, coordination, jumping, and reaching. In addition to participating in PA, children with poor gross motor skills, may also have difficulty sitting in an alert position to watch classroom activity and writing on a blackboard. As a result, the second greatest percentage of time within the program is allocated toward gross motor development (33%) and 17% is devoted to creative play. On a daily basis, daycare providers will be asked to track facilitated physical activities using a simple pre-developed program tracking log sheet. Bi-monthly follow-up support or ‘booster’ sessions will also take place during regular hours within intervention daycare centres. These will be multi-dimensional, and include i) in-centre, ABC project staff-guided, structured PA sessions engaging both preschoolers and providers, ii) goal setting and iterative action planning regarding intervention delivery, and iii) performance monitoring and feedback related to implementation successes and, where relevant, overcoming barriers, troubleshooting, and problem solving.

#### Home environment

While children who attend licensed daycare centres often spend the majority of their waking hours in these environments, the importance of the home environment cannot be overlooked since parents are primary role models and therefore carry considerable influence in the development of their children’s behaviour. Thus, those daycare centres assigned to the DC + HOME arm will, in addition to the in-centre ABC intervention, involve parental/guardian engagement in the promotion and incorporation of daily PA. Parents of preschoolers in this intervention arm will be given the option of participating in two online training sessions known as ‘webinars’ or be given hard copies of the training material. Similar to the provider sessions, the first parental session will be designed to increase awareness with regard to the importance of PA and highlight the dangers in falling prey to the commonly held belief that all preschoolers are vigorously active for a great portion of the day. The second session will expose parents to a variety of activities and provide practical tips and identify opportunities in which PA could be incorporated into a daily routine as well as demonstrate some simple games or play scenarios for small spaces, limited equipment, and outdoor play. Parents will be expected to login, via computer, to participate in these webinars, at their convenience, over the first week of the implementation of the intervention. A short and simple online questionnaire will be provided at the end of the webinars to ensure that the parents have completed the training and acquired the requisite knowledge to implement the home component of the program. Additionally parents will be provided with an ABC Child Activities Booklet outlining fun and simple physical activities to do with their children that require limited equipment. Parents will also receive bi-weekly postcards in the mail which will resemble materials from the *BusyBodies Activity Booklet* produced by the Ontario Public Health Association [55]. Our intent is to identify whether engaging parents is feasible and whether or not they will become involved in promoting PA during the time they spend with their children, and whether their involvement results in children’s increased PA compared to the DC only group.

#### Comparison

Participating daycare centres that are randomized to the wait list control will continue to provide their regular curriculum during the study period. All comparison centres will be offered the ABC trial staff training and resources after the completion of data collection.

The primary outcome (MVPA) will be measured at baseline and at 3-months and 6 months post-intervention. The trial assessments and timeline are illustrated in Table 1. The baseline measurement will occur 2 weeks before the workshops to providers and parental webinars. This will include an assessment of PA and sedentary behaviour, body composition, fundamental and gross motor movement skills, and an assessment of the physical environment in daycare centres and homes as perceived by providers and parents, as well as attitudes, control beliefs, quality of life, perceived competency and intentions of providers and parents toward incorporating PA into the daycare curriculum and home environment, respectively. Immediately following the workshop and webinar training, attitudes, control beliefs, perceived competence, and intentions toward incorporating PA into the daycare setting and home environment will be measured. The parents receiving webinar training will be given an on-line ‘test’ to make sure they understood the training material. The components of the theory of planned behaviour, plus an assessment of any physical changes to the daycare or home environment (perceived by the provider or parent) will also be assessed at 6 months to determine the extent to which they predict PA at different time points. The 3-month evaluations will include children’s PA and sedentary behaviour, body composition and movement skills, as well as an evaluation of quality of life. The 6-month evaluation will include all of the 3-month measures and questionnaires, in addition to evaluations of the physical environment and theory of planned behaviour variables to examine associations with children’s PA.

**Table 1:** **Outcome assessments**

Activity Begins in Childhood (ABC)											
	Group 1: DC				Group 2: DC + HC				Group 3: COM		
	B	PW	3 mos.	6 mos.	B	PW	3 mos.	6 mos.	B	3 mos.	6 mos.
Consent Form	√				√				√		
Socio-Demographic Questionnaire	√				√				√		
Physical Activity Monitor Log	√		√	√	√		√	√	√	√	√
Environment Questionnaires					√		√	√			
EPAO	√		√	√	√		√	√	√	√	√
Gross Motor Assessment	√		√	√	√		√	√	√	√	√
Anthro Measures & Body Composition	√		√	√	√		√	√	√	√	√
Workshops/Webinar	√				√						
Workshop/Webinar Questionnaires	√	√		√	√	√		√			
Quality of Life Questionnaires	√		√	√	√		√	√	√	√	√
Pregnancy and Lifestyle Questionnaire	√				√				√		

B, baseline; mos, months; PW, post workshop; EPAO, Environment and policy assessment and observation.

### Assessment

#### Primary outcome variable

**Physical activity level** Physical activity levels of the preschoolers will be measured using omni-directional Actical^®^ accelerometers (mini Mitter Co., Inc., Bend, OR, USA). At each measurement period (baseline, 3- and 6-months post-workshop intervention), children will wear these activity monitors for a 7-day period. Study staff will train and assist the daycare providers and parents in correct placement of the accelerometer at the child’s time of arrival at the daycare centre on day 1. Data will be collected in 15-second epochs and Adolph et al.’s cut points [56] for preschool-aged children’s PA intensity will be applied to derive time spent at various intensities of movement (e.g., sedentary, light, moderate, vigorous) in harmony with the Canadian Health Measures Survey approach [57]. For ease of comprehension and comparison with other studies, activity data will be summarized and reported as activity minutes per hour, computed from tallied counts for each activity level mean across wear time. In line with current [57–59] and ongoing research in this population, [60] children with ≥5 hours of accelerometer data per day on at least 3 days will be included in the analyses and data will be adjusted for wear time.

### Secondary outcome variables

#### Fundamental/gross motor skills

The test of Gross Motor Development-2 (TGMD-2) will be used to evaluate the effects of the intervention on children’s movement skills [61]. The TGMD-2 is a validated standardized norm-referenced measure of 12 common gross motor skills of children ages 3 to 11 years [62]. The TGMD-2 evaluates 12 gross motor skills divided into two subtests: 1) locomotor (run, hop, gallop, leap, horizontal jump, and slide) and 2) object control (ball skills such as striking a stationary ball, stationary dribble, catch, kick, overhand throw, and underhand roll). This test will be conducted on participating children at baseline and 3- and 6-month assessments.

#### Anthropometrics

Height, weight, body mass index, lean body mass, fat mass, and percent body fat will be measured. Height will be measured using a portable stadiometer (Seca GmBH & Co Kg, Hamburg Germany). Body weight will be assessed using a Tanita scale (Tanita 300-A, Tanita Corporation of America, Inc., Arlington Heights, IL, USA). Body mass index (kg/m^2^) and body composition (lean body mass, fat mass, percent body fat) will be assessed using a RJL Quantum IV Bioelectrical Impedance Analyzer system (RJL Quantum IV, RJL Systems, Clinton Twp, MI, USA, 48035), which accommodates the small feet of young children and has been validated for preschool-aged ranges [63, 64]. This assessment takes about 3 minutes and will be conducted at baseline and 3- and 6-months post-intervention. Body composition measurement will be attempted at the same time of day in all measurement periods in attempt to control for liquid and food intake.

#### Questionnaires

In addition to a simple baseline demographics questionnaire, we will ask all parents about their child’s involvement in extra-curricular activities (sport, dance, music, art playgroups), and potential behaviour change at home (which will be validated by accelerometer data) so that we can account for participation in the analyses. We will collect information about the child’s health-related quality of life using the PedsQL^TM^ Measurement Model for the Pediatric Quality of Life Inventory instrument. The PedsQL^TM^ Measurement Model is a modular approach to measuring health-related quality of life from a multidimensional standpoint (physical, social, emotional, and school functioning) in children and adolescents. The tool is practical as it takes 4 minutes to complete and is reliable (internal consistency for the Total Scale Score: alpha = 0.88 Child Self-Report; alpha = 0.90 Parent Proxy-Report) [65].

The physical childcare environment will be rated using the relevant items from the validated Environment and Policy Assessment and Observation (EPAO) instrument. Construct and predictive validity of the physical activity environment domains, as well as inter-observer reliability of the EPAO instrument have been published previously [66–69]. The PA child behaviours portion of the EPAO instrument, created to evaluate a US-based healthy active living childcare program, is easily applied in the Canadian setting as it assesses the accessibility of the physical environment and facilities within the daycare setting that may promote or restrict PA.

Intervention daycare providers and parents will complete a questionnaire prior to the workshop/webinar training that assesses attitudes, control beliefs, perceived competence, and intentions, as well as support for increasing PA based on the Theory of Planned Behaviour [70, 71]. Following the workshop they will complete another that focuses on their understanding of the central health messages associated with ABC program.

#### Cost analysis

A cost analysis will also be conducted to evaluate the costs of delivering the ABC intervention with the outcomes achieved to provide a valuable analytical framework to guide decision making by those who are responsible for allocating resources.

### Process evaluation

Investigators will measure intervention implementation, defined by Fixsen et al. [72] as the “*specific set of activities designed to put into practice an activity or program of known dimensions*”. Experts in implementation measurement [73–75] have identified a set of key measures and for ABC Trial purposes we will focus on: i) fidelity – comparing actual program component delivery to planned delivery (i.e., workshops**/**webinars delivered/attended**/**accessed online by parents*,* booster sessions delivered as scheduled, postcards delivered to parents as scheduled, site observations, environmental assessment, use of resources and materials); ii) dose delivered by daycare providers (i.e., daily activity checklist, site observations, provider engagement during booster sessions, environmental assessment) or dose received by children (i.e., site observations, environmental assessment, PA assessment on preschoolers); iii) the quality of program delivery (i.e., site observation, environmental assessment, booster session feedback); iv) responsiveness of program participants (i.e., observations); and v) adaptations made to program during implementation (i.e., responsive to daycare provider feedback by incorporating online and hard copy materials, providing additional contact with providers, more example-based workshop material, incorporate FUNdamentals framework). Additionally, we will assess perceived competence, control beliefs, and intentions of both daycare providers and the parents who are involved in the Home Component. The process evaluation methods will include both direct (i.e., EPAO, Actical^®^) and indirect methods (i.e., daily activity checklist). Data from all of these sources will be pooled to assess fidelity and completeness of the implementation in both the daycare and home environment (for participants who receive the Home Component). Further, on a bi-monthly basis, the Master Trainer and Research Coordinator conduct semi-structured interviews with daycare providers to assess any potential barriers/obstacles to implementation of the ABC programming.

### Sample size

The proposed study design is a cluster-randomized trial, where individual preschoolers are nested in daycare centres (clusters). Because observations within clusters are expected to be correlated, the total number of preschoolers that need to be enrolled is larger than would be the case if it were feasible to randomize them individually. This is known as the design effect for a cluster-randomized trial, and it depends on the cluster size(s) and the intra-cluster correlation (ICC). The sample size, therefore, should be multiplied by the design effect (D) [76] (i.e., D = 1 + (m–1) × ICC, where m is cluster size and ICC is the intra-cluster correlation coefficient of the outcome measure).

Licensed daycare centres in the NCR typically enroll between 10 and 40 preschool children, depending on the size of the centre and number of staff. To be conservative, the assumption is that there is an average of 15 preschoolers from each daycare. The estimate of ICC used for the present study was obtained from a similar cluster-RCT, exploring the effectiveness of an elementary school-based PA program aimed at impacting body fat, fitness, and PA, which reported an ICC for MVPA of 0.08. [77] The sample size calculation was performed using the sample size calculator for cluster randomized trials of Campbell et al. [78]. For the primary comparison between the daycare group and the control group, a total of 12 daycare centres (6 per group) is required to achieve a power of 80% to detect a difference between groups in MVPA of 15 min/day with the probability of type-I error fixed at 5%. Since a third group, DC + HOME, will also be studied, an additional 6 daycare centres will be enrolled, for a grand total of 18 daycare centres. The difference of 15 minutes/day was selected for two reasons; i) we felt it clinically relevant given the recent systematic review by Janssen et al. [79] indicating that health benefits may be achieved with an average of 30 min of MVPA/day (at least in elementary school children) and thus an additional 15 minutes on top of what was reported in Canadian preschoolers in a family care setting [33] would be close to 30 min/day, and ii) based on the evidence supporting that an additional 60 minutes per week of PA has been associated with improved aerobic fitness [19] and motor skills [19, 22, 44].

### Statistical analysis

The primary outcome measures will be differences between groups in overall PA and time spent in MVPA, a level of activity associated with health benefits. Secondary outcomes include differences in children’s time spent in sedentary behaviour (objectively measured by Actical accelerometer), height, weight, and body composition (body mass index, lean body mass, fat mass, and percent body fat). In addition, examination to determine if there are group differences in fundamental and gross motor skills will be conducted. Finally, daycare providers’ and parents’ attitudes, control beliefs, perceived competence (ability), and intentions toward incorporating PA into the daycare curriculum or home environments, will be assessed before and after the training workshops/webinars, as well as at 6-months post-workshop intervention.

#### Descriptive analysis

Baseline characteristics of the children and the daycare centres will be summarized descriptively. Categorical variables will be summarized using frequencies and percentages. Continuous variables will be summarized using means, standard deviations, medians, interquartile ranges, and ranges.

#### Primary analysis

To account for the cluster randomized design, a linear mixed effects model with a random effect for daycare will be used to compare time spent per day in MVPA at the 6-month follow-up between the daycare group and the control group. Potentially important child-level covariates will be included, namely age, sex, and participation in organized extra-curricular physical activities (dance, swimming, sport). Here, and throughout, a two-sided *P* value less than 0.05 will be deemed to be statistically significant. Analysis will follow the intention-to-treat principle, that is each daycare—and the children within that daycare—will be included in the group to which they were randomly assigned regardless of the adherence to the intervention.

#### Secondary analyses

To account for PA at baseline, an analysis of covariance modelling approach will also be used (again including a random effect to account for clustering). These analyses will be repeated for the other outcome measures: time spent in sedentary behaviour, motor skills (measured by the TGMD-2)*,* anthropometrics (height, weight, body mass index, lean body mass, fat mass, percent body fat), physical environment (EPAO), and questionnaire data (attitudes, control beliefs, perceived competence, and intentions to increase PA). These analyses will be repeated for the same outcomes at the follow-up times. Predictive models for change in PA based on the questionnaire measures will also be developed. For all models, parameter estimates and 95% confidence intervals will be reported.

Specific to the cost analysis, the data of cost would include all the relevant costs of designing, implementing, monitoring, and analysing the RCT (labour/management cost, research cost, and capital cost). The cost effectiveness of the ABC intervention will be estimated using methods consistent with the guidelines established by the Panel on Cost-Effectiveness in Health and Medicine [80]. We will project costs as well as gains in both life-years and Quality-Adjusted Life Years (QALY) associated with the ABC intervention and with the no-intervention scenario. Health-related utility will be measured using the validated EuroQol five dimensions questionnaire (EQ-5D^TM^) index [81–83], which is a widely used questionnaire for calculating quality-adjusted life-years for assessing cost-effectiveness in healthcare.

Consistent with the panel’s recommendations, the societal perspective will be adopted, and future costs and benefits will be discounted to the present at an annual rate of 3%. The average relative performance of the ABC intervention will be assessed compared to no intervention, using a ratio of the additional expected cost of the program divided by the additional expected QALYs gained relative to the no-intervention alternative.

In addition to the intention-to-treat analyses, we will conduct per-protocol analyses including only preschool children from those daycare centres that were adherent. Evidence suggests that a compliance rate of about 60% is the threshold for achieving specified outcomes [73] and thus a conservative approach will be taken, defining adherence as those who reported complying to at least 70% of the intervention programming as measured by their log sheets.

## Discussion

In keeping with the settings-based approach to health promotion, which acknowledges the influence of place on behaviour [84], it is believed that powerful influences on children’s PA levels are the social and physical environments in which they spend time [67, 85, 86]. As such, the paid child daycare setting provides an ideal opportunity to emphasize the adoption of a physically active lifestyle by enhancing the PA behaviours and movement skills of preschool-aged children which may mitigate the decline in activity often seen during the transition from childhood to adolescence [87]. There is a body of evidence that suggests that in group preschool and child care settings, policies and practices strongly influence children’s PA [67, 86, 88]. However, the efficacy of interventions, especially RCTs, in daycare centre settings on preschool children’s PA and inactivity behaviours has not been thoroughly investigated in Canada.

### How will the results of this trial be used?

At present, the effectiveness of the ABC intervention will rest in our ability to transfer knowledge to, and enhance utilization of, the innovation to daycare-providers who control the child care environment in which children spend time. Despite varying definitions, the measure of effective knowledge transfer or exchange is knowledge utilization [89], namely the uptake and implementation of innovations (evidence-based practices) by decision-makers and practitioners. Our ultimate goal is to initiate and support change in childcare regulatory agency policies that influence preschool programming to ones that foster healthy active behaviour. To do so, the training and education (e.g., degree programs, certification) of early childhood educators would need to be modified to ensure they have the required knowledge and skills for promoting and engaging young children in physical play, reducing sedentary time, and facilitating motor skill development.

## Trial status

Baseline data were collected for the initial cohort in April 2013. The 6-month follow-up data were collected in October, 2013. The second cohort was randomized in November of 2013 with 6-month follow-up taking place in May 2014. Randomization of a third cohort is planned for September 2014.

## Abbreviations

ABC: Activity begins in childhood
COM: Comparison
DC: Daycare
EPAO: Environment and policy assessment and observation
ICC: Intra-cluster correlation coefficient
MVPA: Moderate to vigorous physical activity
NCR: National Capital Region
PA: Physical activity
QALY: Quality-adjusted life years
TGMD-2: Test for gross motor development-2.
